# Development and therapeutic applications of nitric oxide releasing materials to treat erectile dysfunction

**DOI:** 10.4155/fso.15.53

**Published:** 2015-08-01

**Authors:** Kelvin P Davies

**Affiliations:** 1 Professor of Urology, Professor of Physiology & Biophysics, Albert Einstein College of Medicine, Forchheimer 742, 1300 Morris Park Ave, Bronx, NY 10461, USA

**Keywords:** erectile dysfunction, ED, nanoparticles, nitric oxide, NO, phosphodiesterase-5 inhibitors, PDE5 inhibitors

## Abstract

The role of nitric oxide (NO) in erectile physiology is well documented. NO activates relaxation of corporal cavernosal smooth muscle tissue resulting in increased blood flow into the penis resulting in an erection. At present, pharmacologic treatments for erectile dysfunction, such as the phosphodiesterase-5 inhibitors, potentiate the erectile response generated by NO. However, a new class of treatments at a preclinical stage may allow localized delivery of NO to the penis via cutaneous application. These treatments may be of particular value to patients with a neurogenic component to their erectile dysfunction, and may act synergistically with phosphodiesterase-5 inhibitors to increase their efficacy.

**Figure 1.**
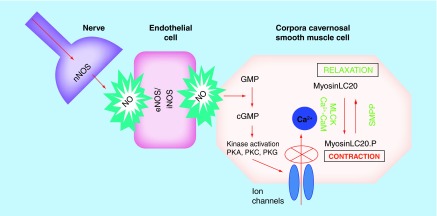
**Nitric oxide pathways involved in erectile physiology.** The initiation of penile erection is controlled by the parasympathetic and sympathetic branches of the autonomic nervous system. Nerve stimulation activates the release of NO from nNOS. This then initiates a cascade effect, activating NO production in endothelial cells through eNOS and iNOS. NO then activates guanylate cyclase, which induces corporal smooth muscle relaxation by increasing intracellular cGMP, which primarily through activation of potassium channels inhibits calcium entry into the cell thereby decreasing intracellular calcium concentrations. Intracellular calcium is the prime determinant of the activity of MLCK. With lower calcium levels in the cell, the predominant direction of myosin is toward dephosphorylation (mediated though MLCK), which leads to smooth muscle relaxation. eNOS: Endothelial nitric oxide; iNOS: Inducible nitric oxide; MLCK: Myosin light chain kinase; NO: Nitric oxide; nNOS: Neuronal nitric oxide synthase; NOS: Nitric oxide synthase.

**Figure 2.**
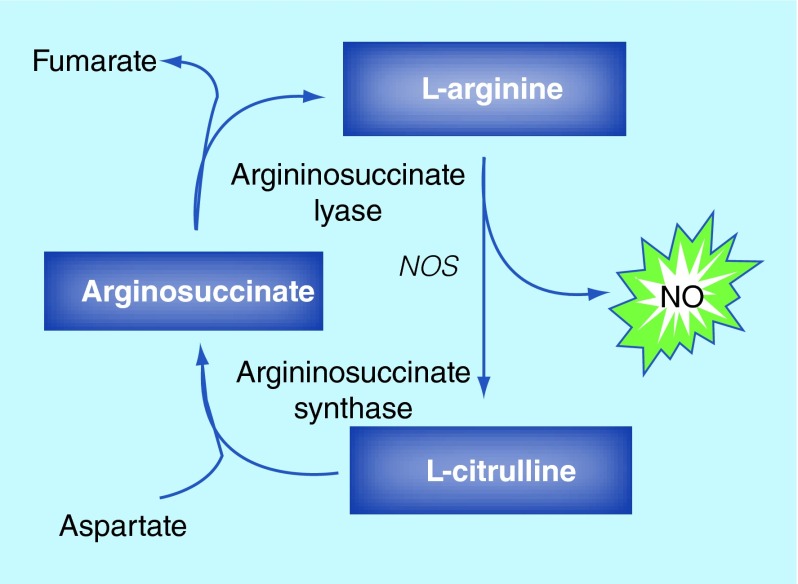
**The metabolism of nitric oxide generating substrates which have been investigated for their ability to treat erectile dysfunction.** NO: Nitric oxide; NOS: Nitric oxide synthase.

**Figure 3.**
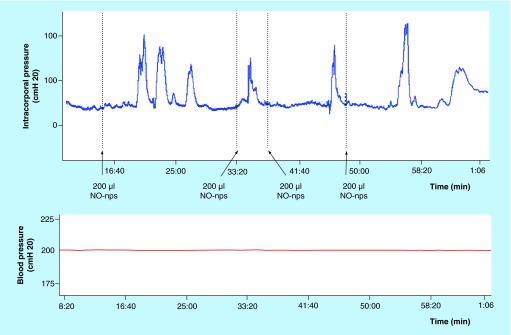
**Example of a continuous trace of intracorporal pressure (upper panel) and systemic blood pressure (lower panel) over the course of an experiment following administration of 200 μl NO-nanoparticles performed topically on the rat penis.** The time points of application of the NO-nanoparticles are shown by the arrows. NO: Nitric oxide. Reproduced with permission from [24] © Wiley (2010).

## Erectile dysfunction as a disease

Erectile dysfunction (ED) is defined as the inability of a man to achieve or maintain an erection sufficient for satisfactory sexual performance [1]. Although considered primarily as a disease affecting the ‘quality of life’ of a patient, ED is not only a physiological condition but also is associated with low self-esteem and deterioration in partner relationships [2,3]. Depending on the cause, ED can be broadly classified as organic, psychogenic or mixed. Psychogenic impotence is where an erection or penetration fails due to thoughts or feelings (psychological reasons) rather than physical pathology. Until the late 1960s, psychogenic reasons were thought to be the cause of the majority of cases of ED. However, following the development of surgical interventions in the 1950s, and pharmacological treatments in the 1990s that were able to successfully treat ED, this position has been totally reversed. Physiological factors are now considered to be the cause of ED in greater than 80% of patients. Two of the most common risk factors for organic ED are diabetes and senescence. Diabetic men are three-times as likely to develop ED as nondiabetic men, and men aged 50–90 years have a ten-times greater risk for ED than those younger than 50 years.

## The role of NO in erectile physiology

The role of nitric oxide (NO) in regulating vascular smooth muscle tone is well documented. Given that erectile physiology is dependent on increased blood flow into the penis through relaxation of the corpora cavernosal smooth muscle tissue, it is not surprising that NO plays an important role in the process [4]. The involvement of NO in eliciting an erection is depicted in Figure 1. The initiation of penile erection is controlled by the parasympathetic and sympathetic branches of the autonomic nervous system [5]. Nerve stimulation activates the release of NO from neuronal nitric oxide synthase (nNOS) [6]. This then initiates a cascade effect, activating NO production in endothelial cells through endothelial and inducible NOS (eNOS/iNOS). Nitric oxide then activates guanylate cyclase, which induces corporal smooth muscle relaxation by increasing intracellular cGMP, which primarily through activation of potassium channels inhibits calcium entry into the cell thereby decreasing intracellular calcium concentrations. Intracellular calcium is the prime determinant of the activity of myosin light chain kinase. With lower calcium levels in the cell, the predominant direction of myosin is toward dephosphorylation (mediated though myosin light chain phosphatase), which leads to smooth muscle relaxation. NO appears to have two roles in the development of an erection: a rapid, brief, calcium-dependent activation of nNOS initiates the erectile process, whereas PI3K/Akt-dependent phosphorylation of eNOS results in sustained NO production and thereby enables full erection attainment [7,8]. It is also possible that increased blood flow into the penis further stimulates NO production from nitrite [9].

Although NO is recognized as playing a central role in erectile physiology, until recently there were no therapeutics that could deliver NO locally. Therefore, most pharmacologic treatments of ED have focused on increasing the effect of NO that is generated in corporal tissue, or to increase the ability of this tissue to generate NO.

## Oral PDE5 inhibitors

At present, the most commonly prescribed treatment for ED are the oral phosphodiesterase-5 (PDE5) inhibitors [1]. As shown in Figure 1, the pathways activated by NO that lead to an erection rely on elevating cellular cGMP levels. However, counteracting the activity of guanylate cyclase are phosphodiesterases which hydrolyze cGMP. In the corpora cavernosal smooth muscle tissue, PDE5 is overexpressed compared with other tissues. When a man is sexually aroused, cGMP synthesis in penile vascular smooth muscle increases and accumulates in healthy individuals; if a PDE5 inhibitor is present cGMP accumulation will be enhanced in the penile tissues, leading to heightened relaxation of corporal smooth muscle tissue. An alternative approach to raising cellular cGMP levels is to utilize guanylate cyclase activators, although at present this remains at an experimental phase [10].

In 1988, sildenafil (Viagra^®^, Pfizer, NY, USA) became the first orally administered PDE5 inhibitor to be approved by the US FDA for the treatment of ED. This was followed by tadalafil (Cialis: 2003), vardenafil (Levitra: 2003) and avanafil (Stendra: 2012). The use of PDE5 inhibitors in patients are overall well tolerated with few side effects. Although effective in approximately 80% of men, a considerable subpopulation of patients remains refractory to this therapy. In particular, patients with a neurogenic component to their development of ED, such as those undergoing prostatectomy, with spinal cord injury or diabetics are particularly refractory to PDE5 inhibitors. This is because neuronal stimulation is necessary for the action of PDE5 inhibitors. Therefore, several experimental approaches to treat ED are being taken that would act upstream of the action of the PDE5 inhibitors, generally targeted to increase NO production.

## Arginine supplementation to treat ED

As shown in Figure 2, L-arginine is the substrate for production of NO by NOS, and there have been several studies in patients and animal models to determine if arginine supplementation can improve ED. Although studies in animals suggested improved erectile function, the level of efficacy in patients chronically administered arginine remains uncertain [11]. However, a recent study where oral arginine was combined with AMP showed some efficacy in patients with mild to moderate ED [12]. Promising results have also been obtained in patients and animal models which are treated with L-citrulline. As shown in Figure 2, L-citrulline can be converted to L-arginine and has the potential advantage that it neither undergo first pass metabolism nor it is metabolized by intestinal bacteria. In rats, L-citrulline supplementation was shown to increase penile levels of NO and improve erectile function [13] and in humans, it was shown to improve the Erection Hardness Score in mild ED patients in a single-blind study [14].

## Gene therapy to increase NOS expression

At least in animal models the potential of overexpression of NOS by gene therapy has shown to be effective in treating animal models of ED. Adenoviral vectors expressing several of the NOS isoforms (eNOS, nNOS, iNOS) when injected into the penis have been shown to increase both cGMP formation and erectile response in both aging and diabetic models of ED [15,16]. Another approach has been adopted to use intracorporal injection of siRNA against the protein inhibitor of NOS (PIN). This approach ameliorated ED in the aging rat [17]. The application of gene therapy to a benign urological disease such as ED, with the danger of germ-line transmission has raised ethical issues which could limit the use of these types of treatments for ED. However, at least a gene therapy treatment has been approved for clinical trials in patients [18].

## Direct delivery of NO

Although NO is recognized as a key player in initiating an erection [19,20], it is a gas and therefore, until recently, it was not technically feasible to directly cause an increase in NO levels in corporal tissue. In general, the direct NO treatment of ED would require topical application of compounds that will generate sufficient NO in the corporal tissue to initiate an erection. Early attempts in the mid to late 1990s focused on topical application of nitroglycerin [21]. Studies demonstrated that application of nitroglycerin paste could result in a greater increase in penile circumference than a placebo, suggesting there was increased blood flow in the penis. However, this did not correspond to an erection sufficient for satisfactory sexual performance, perhaps because effective local levels of NO were not reached, and there are no recent reports detailing the use of this compound in treating ED. Several more recent reports have identified technologies capable of delivering NO to corporal tissue. Studies in the laboratory of Burnett [22] have shown that a sustained NO release compound, abbreviated to C6’, when surgically implanted into the bulbospongiosus muscle at the base of a mouse penis generated local increases in NO with the potential to attenuate priapism in mouse models. Studies in the laboratory of Khera [23] have shown that a long-acting NO-releasing polymer when injected into corporal tissue of diabetic animals improved the erectile response following stimulation of the cavernous nerve and improved the efficacy of PDE5i in the same animals.

A more recent approach to deliver NO topically, pioneered by researchers at Einstein (Dr Davies and Dr Friedman) has been through the use of skin penetrating nanoparticles that can release NO over time (NO-nanoparticles [nps]) [24,25]. The NO-nps have been tested for their ability to restore erectile function in two rat models of ED, age and neurogenic ED. In these models, erectile function is determined by recording the intracorporal pressure/blood pressure (ICP/BP) ratio following topical application of the NO-nps to the penis. As can be seen in Figure 3, approximately 5 min after application of the NO-nps, there were spontaneous increases in ICP/BP. The average peak ICP/BP ratio for the first erection was 0.67 +/- 0.14, corresponding to a full erection in the rat. The erections were typically of less than 2 min duration (average 1.42 min), which is the normal duration of erection in a rat. Control animals, treated with empty nanoparticles did not show erectile activity.

We have extended these studies to a neurogenic model of ED. Male Sprague-Dawley rats underwent cavernous nerve transaction 1 week prior to topical penile application of the NO-nanoparticles [25]. The ICP/BP ratio was followed in anesthetized animals for approximately 2 h. Similar to the aging model of ED shown in Figure 3, five out of eight of these animals had spontaneous erections (ICP/BP greater than 0.6) during the time course of the experiment. Six out of eight animals tested showed an increased ICP/BP ratio from 0.11 +/- 0.01 to 0.25 +/-0.17, demonstrating overall increased corporal blood flow. Control animals treated with empty nanoparticles did not demonstrate any visible erectile response and the ICP/BP ratio was not significantly changed during the course of the experiment.

## Conclusion

NO has a well-defined function in initiating and maintaining an erection. Several materials and approaches that raise local levels of NO in penile tissue have shown promise in treating ED in animal models. Knowledge of the downstream biochemical pathways activated by NO suggest that the efficacy of these NO releasing compounds could be increased when used in combination with PDE5 inhibitors, although this has not as yet been demonstrated experimentally.

**Executive summary**

Oral phosphodiesterase-5 (PDE5) inhibitors are an effective treatment for erectile dysfunction (ED) in most patients.
However, PDE5 inhibitors are ineffective for several subpopulations of patients, particularly those with a neurogenic component underlying their ED. There is therefore a need to develop treatments which target erectile function upstream of PDE5i.
Although nitric oxide (NO) is recognized as playing a significant role in erectile function, until recently, the ability to deliver NO to penile tissues at sufficient levels to result in an erection has proven difficult.
The advent of NO-nanoparticles (NO-nps) which when topically applied to the rat penis with age or neurogenic ED elicit an erection suggests that these compounds may be useful in treating patients.
These compounds act upstream of the PDE5 inhibitors, and therefore combined use of the NO-nps or other releasing compounds and PDE5 inhibitors may act synergistically.
The use of the NO-nps for treating ED is presently at a preclinical stage.
